# 92 Safety & Efficacy of Adipose-derived Stem Cells in Acute Burns: A Randomized Placebo-Controlled Phase-I Trial(ASCAB)

**DOI:** 10.1093/jbcr/irae036.091

**Published:** 2024-04-17

**Authors:** Filip Rangatchew, Linea Melchior, Maj-Lis Talman, Thomas Litman, Jens Kastrup, Krzysztof Drzewiecki, Rikke Holmgaard

**Affiliations:** Rigshospitalet Copenhagen University Hospital, Dept. of Plastic Surgery & Burns, Copenhagen, Hovedstaden; Rigshospitalet Copenhagen University Hospital, Dept. of Pathology, Copenhagen, Hovedstaden; University of Copenhagen, Dept. of Immunology and Microbiology, Copenhagen, Hovedstaden; Rigshospitalet Copenhagen University Hospital, Dept. of Cardiology, Copenhagen, Hovedstaden; Rigshospitalet Copenhagen University Hospital, Dept. of Plastic Surgery and Burns, Copenhagen, Hovedstaden; Department of Plastic Surgery and Burns Treatment, University Hospital of Copenhagen, Copenhagen, Hovedstaden; Rigshospitalet Copenhagen University Hospital, Dept. of Plastic Surgery & Burns, Copenhagen, Hovedstaden; Rigshospitalet Copenhagen University Hospital, Dept. of Pathology, Copenhagen, Hovedstaden; University of Copenhagen, Dept. of Immunology and Microbiology, Copenhagen, Hovedstaden; Rigshospitalet Copenhagen University Hospital, Dept. of Cardiology, Copenhagen, Hovedstaden; Rigshospitalet Copenhagen University Hospital, Dept. of Plastic Surgery and Burns, Copenhagen, Hovedstaden; Department of Plastic Surgery and Burns Treatment, University Hospital of Copenhagen, Copenhagen, Hovedstaden; Rigshospitalet Copenhagen University Hospital, Dept. of Plastic Surgery & Burns, Copenhagen, Hovedstaden; Rigshospitalet Copenhagen University Hospital, Dept. of Pathology, Copenhagen, Hovedstaden; University of Copenhagen, Dept. of Immunology and Microbiology, Copenhagen, Hovedstaden; Rigshospitalet Copenhagen University Hospital, Dept. of Cardiology, Copenhagen, Hovedstaden; Rigshospitalet Copenhagen University Hospital, Dept. of Plastic Surgery and Burns, Copenhagen, Hovedstaden; Department of Plastic Surgery and Burns Treatment, University Hospital of Copenhagen, Copenhagen, Hovedstaden; Rigshospitalet Copenhagen University Hospital, Dept. of Plastic Surgery & Burns, Copenhagen, Hovedstaden; Rigshospitalet Copenhagen University Hospital, Dept. of Pathology, Copenhagen, Hovedstaden; University of Copenhagen, Dept. of Immunology and Microbiology, Copenhagen, Hovedstaden; Rigshospitalet Copenhagen University Hospital, Dept. of Cardiology, Copenhagen, Hovedstaden; Rigshospitalet Copenhagen University Hospital, Dept. of Plastic Surgery and Burns, Copenhagen, Hovedstaden; Department of Plastic Surgery and Burns Treatment, University Hospital of Copenhagen, Copenhagen, Hovedstaden; Rigshospitalet Copenhagen University Hospital, Dept. of Plastic Surgery & Burns, Copenhagen, Hovedstaden; Rigshospitalet Copenhagen University Hospital, Dept. of Pathology, Copenhagen, Hovedstaden; University of Copenhagen, Dept. of Immunology and Microbiology, Copenhagen, Hovedstaden; Rigshospitalet Copenhagen University Hospital, Dept. of Cardiology, Copenhagen, Hovedstaden; Rigshospitalet Copenhagen University Hospital, Dept. of Plastic Surgery and Burns, Copenhagen, Hovedstaden; Department of Plastic Surgery and Burns Treatment, University Hospital of Copenhagen, Copenhagen, Hovedstaden; Rigshospitalet Copenhagen University Hospital, Dept. of Plastic Surgery & Burns, Copenhagen, Hovedstaden; Rigshospitalet Copenhagen University Hospital, Dept. of Pathology, Copenhagen, Hovedstaden; University of Copenhagen, Dept. of Immunology and Microbiology, Copenhagen, Hovedstaden; Rigshospitalet Copenhagen University Hospital, Dept. of Cardiology, Copenhagen, Hovedstaden; Rigshospitalet Copenhagen University Hospital, Dept. of Plastic Surgery and Burns, Copenhagen, Hovedstaden; Department of Plastic Surgery and Burns Treatment, University Hospital of Copenhagen, Copenhagen, Hovedstaden; Rigshospitalet Copenhagen University Hospital, Dept. of Plastic Surgery & Burns, Copenhagen, Hovedstaden; Rigshospitalet Copenhagen University Hospital, Dept. of Pathology, Copenhagen, Hovedstaden; University of Copenhagen, Dept. of Immunology and Microbiology, Copenhagen, Hovedstaden; Rigshospitalet Copenhagen University Hospital, Dept. of Cardiology, Copenhagen, Hovedstaden; Rigshospitalet Copenhagen University Hospital, Dept. of Plastic Surgery and Burns, Copenhagen, Hovedstaden; Department of Plastic Surgery and Burns Treatment, University Hospital of Copenhagen, Copenhagen, Hovedstaden

## Abstract

**Introduction:**

Burns can progress in depth over time due excessive inflammation, which hinders wound healing. Mesenchymal stem cells have demonstrated promising outcomes in preclinical studies due to their immunomodulatory, anti-apoptotic and regenerative properties.

This clinical trial aimed to evaluate safety and effectiveness of culture-expanded allogeneic adipose-derived mesenchymal stem cells (ASCs) in healing of acute burns.

Hypothesis: administering stem cell therapy shortly after the burn injury would: 1) reduce burn-induced inflammation, and 2) enhance wound healing dynamics

**Methods:**

Study design: single-center, phase I, randomized placebo-controlled, double-blind trial on six adult patients with deep partial-thickness burns, each serving as their own control. Two deep partial-thickness burns of uniform size in each patient were randomized to receive dermally injected ASCs and placebo shortly after the burn injury.

The primary outcome was change in wound healing using photodigital planimetric quantification of re-epithelialization on days 5, 10, 14 and 21. Secondary outcomes included: safety by recording adverse events and blood sampling for alloimmunization; change in expression of relevant genes (mRNA) by analyzing biopsies at baseline and day 14; change in blood perfusion by laser Doppler imaging at baseline, intervals of 30 min from 0-120 min, day 5, 10, 14 and 21.

Descriptive statistics were employed for wound healing, blood perfusion and safety outcomes. For biopsy analysis, differentially expressed genes (DEG) were identified by paired t-test (patient eliminated as factor, p< 0.05), log2FC ≥ |1.00|). Statistical significance was set to p< 0.05, and the FDR adjusted p-values (q values) reported.

**Results:**

Deep partial-thickness burns were confirmed histologically. Median time from injury to intervention was 15.1 h (range, 10.1-23.9 h).

The primary outcome showed that burns treated with stem cells vs. placebo improved the wound healing dynamics with 1.5 to 5.5-fold on different timepoints in the wound healing process (range, day 10-48). In two patients wound healing was negatively impacted by early infections, not related to the cell product. Good safety was demonstrated with no instances of adverse reactions. Only one patient had developed donor-specific HLA antibodies. The mRNA expression analysis showed that five DEG were significant in their change of expression as a result of ASCs: IL-6 (diff: -0.6018, p=0.0494), ITGA11 (diff: -0.7033, p=0.0161), CXCR2 (diff: -0.8459, p=0.011), HLA-B (diff: -1.3286, p=0.0448) and EIF4EBP1 (diff: -1.3498, p=0.0388) all q=0.70. There were no stem-cell related effects on blood perfusion.

**Conclusions:**

ASC-therapy for burns was safe and demonstrated potential in improving wound healing dynamics. These results encourage further exploratory and confirmatory trials.

**Applicability of Research to Practice:**

The future perspective is to develop a novel, off-the-shelf, cell-based therapy for burn patients.

